# Relative Efficacy of Quercetin Compared with Benzydamine Hydrochloride in Minor Aphthae: A Prospective, Parallel, Double Blind, Active Control, Preliminary Study

**DOI:** 10.1155/2017/7034390

**Published:** 2017-11-13

**Authors:** Maitreyi Pandya, Anupama N. Kalappanavar, Rajeshwari G. Annigeri, Dhanya S. Rao

**Affiliations:** ^1^Oral Medicine and Radiology, Private Practice, New Delhi, India; ^2^Oral Medicine and Radiology, College of Dental Sciences, Davangere, India; ^3^Oral Medicine and Radiology, A. J. Institute of Dental Sciences, Mangalore, India

## Abstract

**Background and Objectives:**

Recurrent aphthous stomatitis is an inflammatory condition present since ancient era wherein numerous treatment modalities have been tried. But complete eradication of the disease has not been possible and hence newer agents are being introduced. One such agent is a flavonoid named quercetin with proven antioxidant, anti-inflammatory, and ulcer healing properties.

**Methods:**

40 patients with minor aphthous ulcers were divided equally into two groups: A and B. Group A patients were advised to apply quercetin gel and Group B patients were advised to take benzydamine hydrochloride mouth wash. Clinical evaluation including assessment of ulcer size and pain score and questionnaire about the acceptability of both the drugs in terms of taste and ease of application was carried out. Each criterion was compared and statistically analyzed.

**Results:**

There was statistically significant reduction in the mean score of pain sensation and ulcer area in both the groups. Quercetin showed statistically highly significant ulcer size reduction as compared to benzydamine hydrochloride.

**Conclusion:**

From the present study, it is evident that quercetin is safe, well tolerated, and effective therapy which promotes complete ulcer healing in a short duration of time.

## 1. Introduction

*“Medicine Is a Science of Uncertainty and an Art of Probability” (Louis Pasteur)*. Recurrent aphthous stomatitis (RAS) is one of the most common oral mucosal disorders affecting 5–25% of the general population. When specific ethnic, high socioeconomic groups or selected populations were studied, the prevalence could be as high as 50–60% [1, 2]. Patients with recurrent aphthous stomatitis experience oral pain causing discomfort, ranging from simple annoyance to an intensely painful period that interferes with normal oral activities at some time in their lives [3]. Despite extensive investigations, the exact etiology of RAS remains elusive though various factors such as local trauma, familial tendency, nutritional deficiencies, immune disturbances, hormonal imbalance, microbial factors (both bacterial and viral), underlying systemic diseases, medications, stress, and allergy may contribute to the pathogenesis of this clinical entity [1, 2].

Treatment for oral aphthous ulcers includes antibacterial, anti-inflammatory, and analgesic mouth rinses, immunomodulators, hormones, and lasers. In patients with frequent exacerbations or more severe forms of aphthae that are unresponsive to topical treatments, systemic agents such as corticosteroids, colchicines, dapsone, or antibacterials are indicated [4]. However, the treatment of aphthous ulcers remains unsatisfactory, since both topical and systemic therapies provide only palliative care, thereby reducing only the severity of symptoms. Thus, substantial need exists for an effective and well tolerated agent that can promote complete ulcer healing within a short period of time without any side effects.

Quercetin is a naturally occurring flavonoid that has a long history of consumption as part of the normal human diet [5]. Several biological and pharmacological functions have been ascribed to quercetin, including strong antioxidant and anti-inflammatory properties [5]. Quercetin being one of the most powerful flavonoids protects the body against reactive oxygen species, by direct scavenging of free radicals. It also chelates ions of transition metals such as iron which can initiate the formation of oxygen free radicals [6] and inhibits xanthine oxidase activity and nitric oxide induced radical damage, all of which have been implicated in the etiopathogenesis of RAS [6, 7]. Tumor necrosis factor-alpha (TNF-*α*), an important inflammatory mediator and a critical cytokine for adequate host defense which is increased in RAU, is significantly inhibited by quercetin [8], indicating that it has the capacity to modulate the immune response and has potential anti-inflammatory activity [6, 7].

Quercetin also exerts an enzyme inhibitory action on phospholipases which catalyses the release of arachidonic acid from phospholipids stored in cell membranes thereby decreasing synthesis of thromboxane, prostaglandins, and leukotrienes [4, 5]. In addition, quercetin also inhibits the enzymes cyclooxygenase and lipooxygenase which catalyses the conversion of arachidonic acid to its metabolites suggesting it to be a potent anti-inflammatory agent [6]. Also quercetin seems to exert antimicrobial activity against many strains of bacteria, thereby removing the bacterial contamination [6]. The healing properties of quercetin include enhancement of myofibroblast and epithelial cell growth, which are vital to the tissue repair process, as suggested in a number of animal studies conducted for the management of gastric and colon ulcers [4].

Benzydamine HCL is a nonsteroidal anti-inflammatory drug which is devoid of activity on arachidonic acid metabolism [8, 9]. The anti-inflammatory activity of benzydamine has been recently related to its capacity to inhibit the production of proinflammatory cytokines (TNFa, IL-1b), without significantly affecting other inflammatory cytokines (IL-6, IL-8) and, importantly, anti-inflammatory cytokines (IL-10, IL-1ra). It has also been seen that benzydamine inhibits the migration of inflammatory leukocytes and that this effect is associated with inhibition of the mitogen-activated protein kinase (MAPK) pathway. Inhibition of MAPK activation and cell migration in response to chemotactic agents is also likely to contribute to the anti-inflammatory activity of this compound [9]. Studies have proved that benzydamine has general antimicrobial properties with a rapid biocidal activity against a variety of organisms at concentrations less than those advocated for treatment of inflammatory conditions [10]. It has been hypothesized that benzydamine produces local analgesia by stabilisation of the cellular membrane and inhibition of prostaglandin synthesis [11].

Given the adverse effects and limited efficacy of the current treatment modalities, the present study was undertaken to unearth a newer treatment modality by determining the potential healing properties of quercetin as compared to an active control drug benzydamine hydrochloride in otherwise healthy patients presenting with minor aphthous ulcers.

## 2. Materials and Methods

The present randomized, prospective, parallel-group, active controlled clinical study was conducted in the Department of Oral Medicine and Radiology, College of Dental Sciences, Davangere, from 2012 to 2014, wherein 40 patients with characteristic clinical features of minor aphthous ulcers and willing to undertake the treatment until complete healing of the ulcer were included. Patients with major or herpetiform aphthous ulcers, any other associated oral mucosal diseases, systemic diseases affecting healing of ulcers, and deleterious habit history of tobacco in any form and patients on other medications for minor aphthous ulcers were excluded from the study. Institutional ethical clearance was obtained and informed consent was procured from the participants of the study after explaining the entire procedure of the study. Two observers conducted the study, with one dispensing the drug and the other measuring the parameters, thereby preventing bias.

### 2.1. Data Collection

Patients in generally good health, irrespective of the site and gender with RAS, were selected. A diagnosis of aphthous ulcer was made if it occurred in the nonkeratinized mucosa as a shallow crateriform ulcer covered by a whitish yellow pseudomembrane and presented with a round, regular border with a surrounding erythematous halo [12].following data were recorded.

*Number of Ulcers and Duration*. Subjects with <3 ulcers were included for ease of assessment. Diagrammatic representation of each ulcer was done in the proforma.

*Pain Intensity*. It was measured using VAS consisting of a 10 cm line [13], with 0 being no pain and 10 being severe pain.

Aggravating factors like menstruation and stress were also recorded. In addition to this, patients were also questioned about the presence of any joint pain, eye or skin lesions, and gastrointestinal disturbances [1] and general physical examination was carried out to rule out the same.

*Size of Each Ulcer.* A transparent plastic sheet was directly applied on the ulcer and using a permanent water proof marker pen the circumference of the ulcer was traced and then placed on a graph paper and the number of mm^2^ units included inside the area drawn was counted [4]. Pretreatment photographs were taken and the treatment was commenced on the same day with strict infection control measures.

### 2.2. Quercetin Gel: (Table 1)

Hydroxyethyl cellulose and distilled water required for the gel preparation were continuously stirred by mechanical stirrer, till the polymer dissolves, and then the appropriate quantity of quercetin dissolved in small quantity of water was added, followed by methyl paraben which served as preservative, glycerine as a humectant, and peppermint oil as a flavoring agent. The prepared gel was packed in sterile 10 ml aluminum collapsible tubes and the tubes were sealed and labeled.

### 2.3. Drug Administration

40 patients were randomly assigned to 2 groups, A and B. Group A patients received a gel containing approximately 2% quercetin to be applied topically on the ulcers TID for total of 7 days and were restrained from eating, drinking, or rinsing the mouth for 30 minutes after each application [14]. Group B patients received 0.15% benzydamine hydrochloride mouthwash (Tantum oral rinse) to be rinsed for 30 seconds and then expelled out, approximately 15 minutes before meals TID for 7 days [15].

Patients were recalled on 2nd, 4th, and 7th day of the treatment. Ulcer size and pain assessment was done on each visit. Allergic reaction was also monitored. At day 7 a questionnaire about the acceptability of the quercetin and benzydamine hydrochloride in terms of taste and ease of application was provided to the subject. The demographic and clinical examination data so gathered was sorted and tabulated into a master chart which was subjected to appropriate statistical analysis.

### 2.4. Statistical Analysis

Statistical analysis was carried out using SPSS package (version 19). Results were expressed as mean ± SD. One-way ANOVA and post hoc Tukey's test were used for groupwise comparisons. Pairwise comparison was made by paired *t*-test. For all the tests, a *p* value of 0.05 or less was considered as statistically significant.

## 3. Results

An age range of 9–58 years was seen, with the mean age of patients in group A being 25.1 ± 13.79 years and group B being 27 ± 13.29 years. Group A had 14 males and 6 females whereas group B had 12 males and 8 females. The mean duration of occurrence of ulcers in group A was 2.1 ± 0.85 days and group B was 2.1 ± 0.71 days. No statistical significance with respect to gender, age, and duration of the ulcers was seen between both the groups. In group A, 12 (60%) patients showed complete ulcer healing and 8 (40%) patients showed partial ulcer healing on day 7 whereas in group B they were 7 (35%) and 13 (65%), respectively.

**Figure 1:**
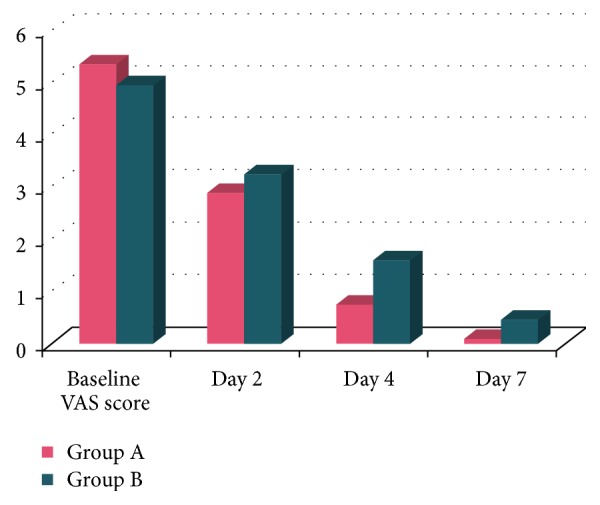
Intergroup comparison of VAS score for pain assessment.

The mean pain score of ulcers at the baseline (1st visit) was 5.35 ± 1.84 for group A and 4.95 ± 1.7 for group B. During the treatment period, the mean pain score at day 2 (2nd visit), day 4 (3rd visit), and day 7 (4th visit) was 2.9 ± 1.71, 0.75 ± 1.29, and 0.1 ± 0.44 and 3.25 ± 1.91, 1.6 ± 2.13, and 0.45 ± 1.19 for groups A and B, respectively. Hence, the mean reduction in pain score from baseline to day 2 was 2.45, from baseline to day 4 was 4.6, and from baseline to day 7 was 5.25 for group A and 1.7, 3.35, and 4.50 for group B. Also, the mean reduction in pain score from day 2 to day 4 was 1.65 and from day 2 to day 7 was 2.8 for group A and 1.65 and 2.8 for group B. The mean reduction in pain score from day 4 to day 7 was 0.65 for group A and 1.15 for group B. All these mean reductions were statistically highly significant (*p* < 0.001) (Figure 1). At the baseline (1st visit) the mean pain scores difference for groups A and B was 0.40. At day 2, the difference was 0.35, at day 4 difference was 0.85, and at day 7 the difference was 0.35. All these were statistically nonsignificant. The mean difference for the pain score from baseline to day 7 was 5.25 in group A and 4.5 for group B which were both statistically highly significant (*p* < 0.001). But, the mean difference for the pain score between groups A and B on day 7 was 0.35 which was not statistically significant (*p* = 0.246).

**Figure 2:**
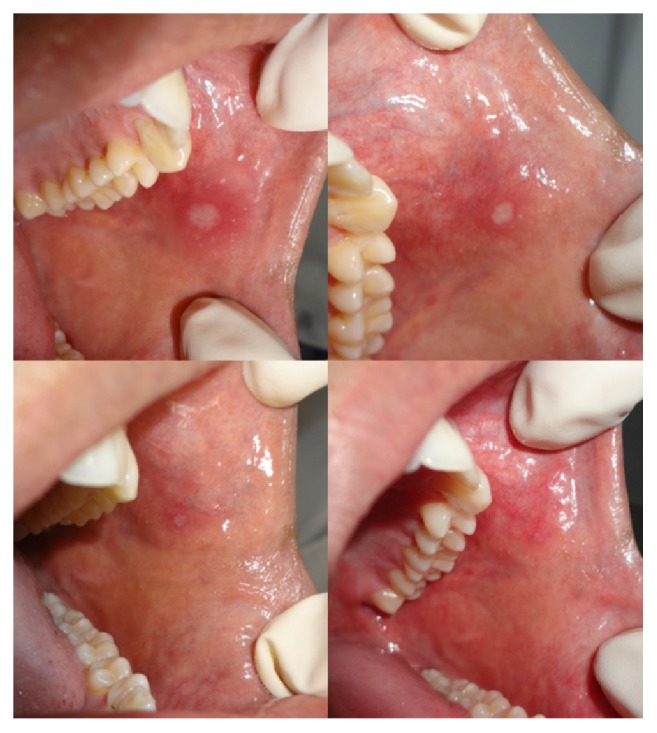
Ulcer healing seen in group A.

**Figure 3:**
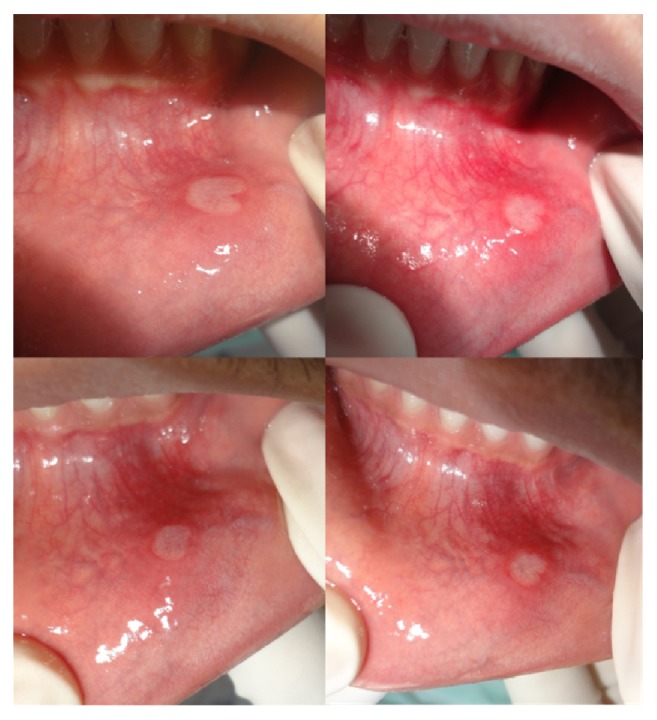
Ulcer healing seen in group B.

The mean score of ulcer area at the baseline for group A was 28.25 ± 6.71 whereas for group B it was 29.7 ± 8.4. During the treatment period, the mean score of ulcer area at day 2, day 4, and day 7 was 15.75 ± 4.5, 6.95 ± 2.91, and 1.3 ± 1.94, respectively, for group A and 23.1 ± 7.6, 13.8 ± 6.7, and 5.9± 6.5, respectively, for group B (Figures 2 and 3). Hence, the mean reduction in ulcer area from baseline to day 2 was 12.5, from baseline to day 4 was 21.3, and from baseline to day 7 was 26.9 for group A and 6.5, 15.9, and 23.8 for group B. Also, the mean reduction in ulcer area from day 2 to day 4 was 8.8 and from day 2 to day 7 was 14.45 for group A and 9.3 and 17.2 for group B. The mean reduction in ulcer area from day 4 to day 7 was 5.65 and 7.9 for groups A and B, respectively. All these mean reductions were statistically highly significant (*p* < 0.001). At the baseline, groups A and B had a mean difference in their ulcer area of 1.45 and it was statistically nonsignificant (*p* = 0.55). At day 2 mean difference was 7.4, at day 4 the difference was 6.85, and at day 7 the difference was 4.6. These were statistically highly significant (*p* < 0.001).

**Figure 4:**
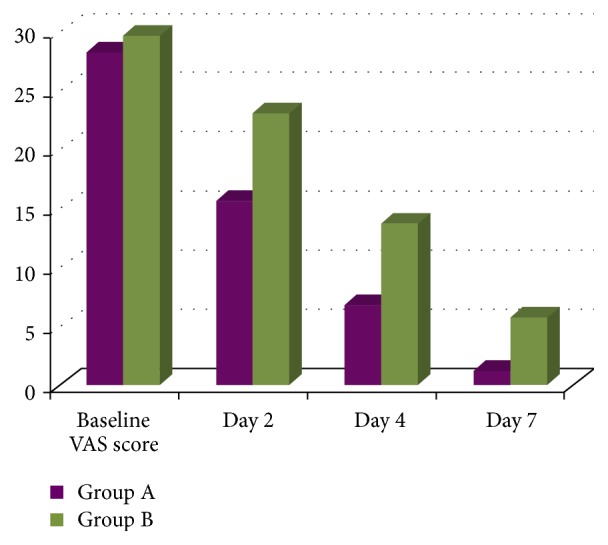
Intergroup comparison of ulcer areas (in mm^2^).

The mean difference for the ulcer size in group A from baseline to day 7 was 26.95 and in group B was 23.8. On intergroup comparison, the mean difference for the ulcer size between groups A and B on day 7 was 4.6 which was statistically highly significant (*p* < 0.001) (Figure 4).

With regard to the taste of the gel, 80% reported good taste and with respect to the ease of application 75% found it easy to apply. With regard to the taste of the mouthwash, 74% found the taste acceptable and, with respect to the ease of application, 80% found it easy to apply.

## 4. Discussion

Aphthous stomatitis has been studied for many years by numerous investigators. Recently, oxidant-antioxidant imbalance has been given prime importance in the etiology of recurrent aphthous stomatitis [16]. Several biological and pharmacological functions have been ascribed to quercetin, including strong antioxidant and anti-inflammatory properties.

60% of patients in group A and 35% patients in group B showed complete ulcer healing on day 7. These results were similar to the study conducted by Mostafa and Ibrahem [4]. The rapid ulcer healing in the quercetin group can be attributable to the anti-inflammatory and antioxidative properties of the drug. Benzydamine, on the other hand, provides only pain relief and does not accelerate ulcer healing [17].

Pain reduction is the cornerstone in the management of RAS patients. In the present study, the study drug quercetin successfully achieved this goal by producing highly statistically significant reduction in pain scores from baseline to day 2, day 4, and day 7 during the treatment period. Similarly the active control drug benzydamine hydrochloride also showed significant pain reduction over the 7-day period, but, on intergroup comparison, there was no statistically significant difference between the two groups with regard to pain reduction. These findings were in contrast to the study conducted by Mostafa and Ibrahem [4] wherein a moderately significant difference (<0.01) was noted between the two groups. The significant pain reduction in group A could be attributable to the fact that quercetin induces an antinociceptive effect primarily by modulating the adrenergic pathways [18]. Quercetin also has a powerful anti-inflammatory action which could be responsible for the analgesia [19, 20]. Also, the rapid healing of the ulcers in this group could account for the significant decrease in the symptoms associated with it. Likewise, in case of the active control group, benzydamine hydrochloride has anti-inflammatory, analgesic, and local anaesthetic properties when applied topically [9, 11]. In a study conducted by Matthews et al. [21], comparing the efficacy of benzydamine HCL to that of chlorhexidine and placebo groups, no statistically significant differences were noted between any of the treatments tested. Similarly in the present study too, no statistically significant difference was noted between the two groups with respect to pain reduction.

In the present study, the study drug quercetin produced highly statistically significant reduction in ulcer area from baseline to day 2, day 4, and day 7 during the treatment period. Similarly benzydamine hydrochloride also showed highly statistically significant reduction in ulcer area over the 7-day period. On intergroup comparison, the mean score of ulcer area at the baseline was statistically nonsignificant (*p* = 0.55). However, statistically highly significant difference was noted between the two groups during the subsequent visits. These findings were consistent with the study conducted by Mostafa and Ibrahem [4] wherein a highly significant difference (*p* = 0.004) was noted between the two groups for ulcer size. This clinical improvement could be due to antiulcerative action of quercetin wherein it enhances myofibroblast and epithelial cell growth, which are vital to the tissue repair process [4]. Accelerated ulcer healing also requires removal of bacterial contamination from the wound to provide favourable grounds for mucosal cell growth and repair [4]. Various studies have shown quercetin has antibacterial property as well [6]. On the other hand, benzydamine hydrochloride provides only palliative care whereby it reduces the pain perception but does not aid in the ulcer healing [16]. Furthermore, quercetin was given in the form of a gel which provided a thick encompassing coat over the ulcer area preventing it from bacterial contamination and mechanical trauma; also the retention period of a gel over the ulcer surface is more than that of a mouth rinse, thus providing prolonged therapeutic benefits.

The ease of application of benzydamine hydrochloride was better than that of the quercetin gel. This could be ascribed to the fact that benzydamine hydrochloride given in the form of a mouth rinse was more accessible to the various areas in the oral cavity as compared to quercetin which was given in a gel form. These results were found to be similar to those of Mostafa and Ibrahem [4] and Roopashri et al. [15] studies.

None of the patients in our study reported any serious side effects due to the study drugs. In the benzydamine hydrochloride group, 8 patients experienced a transient stinging sensation shortly following the rinsing with the drug. In a study conducted by Matthews et al. [21], it was noted that eight patients stated a personal preference for benzydamine because of the local anaesthetic effect of benzydamine, which gave pain relief in spite of the transient stinging sensation associated with its usage. Topical use of high potency glucocorticoids may cause oral pseudomembranous candidiasis and suppression of the HPA axis. In 2.1% of 991 patients treated with topical amlexanox, adverse effects like stinging, dryness, bumps on the lips, and mucositis were noted. Chlorhexidine mouthwash has a bitter taste and causes brown staining of the teeth and tongue. On the contrary, no adverse effects were seen in the study drug quercetin and this was consistent with the study conducted by Mostafa and Ibrahem [4].

Although the results of the study are highly encouraging, there were also a few limitations that were encountered in the present study. So far only one study using quercetin in RAS management has been documented in the literature. The sample size in the present study was small. The application of the medicament to the remote areas of the oral cavity was relatively difficult due to inaccessibility of quercetin in the gel form. The patients with multiple ulcers and major aphthous ulcers could not be included in the study for ease of assessment of the clinical parameters. Furthermore the study involved the comparison of a mouth rinse to that of a gel, which added the confounding factor of comparing two different modes of local drug delivery systems.

Further studies are recommended on a larger sample of patients, over a longer follow-up period, in a larger multicentric setup, and with the evaluation of immunological markers to maximize the sensitivity for detecting subtle changes of the mucosa during the course of the treatment. Quercetin gel is not available commercially as of now; it is recommended that commercial preparation of topical quercetin gel be made available in the near future.

*In conclusion*, the results from our study are highly encouraging; the use of topically applied quercetin gel has shown salutary results compared to benzydamine hydrochloride in minor RAS patients and further research with this drug entity might prove beneficial.

## Figures and Tables

**Table 1:** Ingredient list.

Ingredient	Quantity
Quercetin	2 g
Methyl paraben	0.01 g
Glycerine	4 g
Peppermint oil	0.02 ml
Polymer (hydroxyethyl cellulose)	100 g qs
Water	75 ml approx.
